# Partial Purification and Characterization of the Mode of Action of Enterocin S37: A Bacteriocin Produced by *Enterococcus faecalis* S37 Isolated from Poultry Feces

**DOI:** 10.1155/2010/986460

**Published:** 2010-08-02

**Authors:** Y. Belguesmia, Y. Choiset, H. Prévost, M. Dalgalarrondo, J.-M. Chobert, D. Drider

**Affiliations:** ^1^ONIRIS. Ecole Nationale Vétérinaire, Agroalimentaire et de l'alimentation Nantes-Atlantique, Rue de la Géraudière, BP 82225, 44322 Nantes Cedex 3, France; ^2^UR 1268 Biopolymères Interactions Assemblages, équipe Fonctions et Interactions des Protéines Laitières, INRA, BP 71627, 44316 Nantes Cedex 3, France; ^3^Department of Research into Sanitary Risks and Biotechnology of Reproduction, UPSP 5301 DGER, Nantes-Atlantic National College of Veterinary Medicine, Food Science and Engineering (ONIRIS), ONIRIS La Chantrerie, BP 40706, 44307 Nantes Cedex 03, France

## Abstract

The aim of this research was to purify and characterize the mode of action of enterocin S37, a bacteriocin produced by *Enterococcus faecalis* S37, a strain recently isolated from the chicken feces. Enterocin S37 has a molecular weight comprised between 4 and 5 kDa. It remained active after 1 h at 80^o^C and at pH values ranging from 4.0 to 9.0. Furthermore, cell-free supernatant of *Enterococcus faecalis* S37 and purified enterocin S37 were active against Gram-positive bacteria including *Listeria monocytogenes* EGDe, *L. innocua* F, *Enterococcus faecalis* JH2-2, and *Lactobacillus brevis* F145. The purification of enterocin S37 was performed by ammonium sulfate precipitation followed up by hydrophobic-interaction chromatography procedures. Treatment of enterocin S37 with proteinase K, *α*-chymotrypsin, and papain confirmed its proteinaceous nature, while its treatment with lysozyme and lipase resulted in no alteration of activity. Enterocin S37 is hydrophobic, anti-*Listeria* and likely acting by depletion of intracellular K^+^ ions upon action on K_ATP_ channels. This study contributed to gain more insights into the mode of action of enterocins.

## 1. Introduction

Bacteriocins are ribosomally synthesized antimicrobial peptides produced by bacteria that deserve considerable interest for their use as natural and nontoxic food preservatives as well as for their potential use in human and veterinary applications, and in the animal production field [1]. In 1976 Tagg et al. defined bacteriocins as proteinaceous compounds that kill closely related bacteria but it is now recognized that bacteriocins can also have activity across genera (broad spectrum) [2–4]. The antimicrobial sensitivity of a target strain to any given bacteriocin may depend on the ecological conditions under which it is grown as variations in salt concentration, pH, the presence of membrane disrupting molecules, or inducing cultures, and a large number of other environmental parameters can have a significant impact [5, 6]. The classification of Gram-positive bacteriocins is rendered difficult because of their heterogeneity and thus, as the number of Gram-positive bacteriocins identified has continued to increase, classification schemes have had to continuously evolve [1].

Klaenhammer [8] suggested four classes of bacteriocins including class I or lantibiotics, which are small membrane-active peptides (< 5 kDa) containing the unusual amino acids lanthionine or *β*-methyl lanthionine (hence the name lantibiotics) and dehydrated residues. Nisin is the most characterized of the class I bacteriocins. Class II is defined as small heat-stable nonlanthionine containing membrane active peptides characterized by the presence of a Gly-Gly processing site in the bacteriocins precursor, the presence of amphiphilic helices with varying amounts of hydrophobicity and moderate to high-heat stability. These were further subdivided into three subgroups named subclass IIa, which are *Listeria* active peptides and YGNGV box in the N-terminal [9]; subclass IIb: poration complexes consisting of two proteinaceous peptides for activity [10] and subclass IIc, which contains thiol activated peptides requiring reduced cysteyl residues for activity [8]. Class III is large heat labile proteins, often with enzymatic activity and finally class IV, which is composed of complex proteins. However, recent classification reported only three major classes [11].

Many *Enterococcus* strains from various ecosystems were characterized as antagonists of broad range of foodborne pathogens [12, 13]. The bacteriocins produced by enterococcal strains displayed heterogeneous structures and differences in their mode of action [14]. Most of bacteriocin Producers enterococcal strains were isolated not only from different types of food, but also from broilers [2, 4] and animal secretions [15]. As reported in the literature, some of these bacteriocins exhibited high activities against broad spectrum of bacteria including Gram-positive bacteria, like *Staphylococcus* and *Streptococcus* and Gram-negative bacteria like *Campylobacter *or* Salmonella*, strains of medical interest [2, 4, 16]. The ability to produce such a biocompound may play a role in providing an ecological advantage on nonbacteriocin producer strains. Previously, we have isolated a strain of *E. faecalis* from chicken drop [7] which appeared to be a bacteriocin producer. In this work, we have undertaken the purification and characterization of the mode of action of this peptide named enterocin S37.

## 2. Materials and Methods

### 2.1. Bacteriocin Producer Strain

*E. faecalis* S37 was isolated from poultry feces collected in a French farm located in Ancenis (West of France) [7]. *E. faecalis* S37 was identified by sequencing whole 16S rRNA gene [7]. *E. faecalis* S37 showed in first instance an antagonism directed against strains belonging to *Listeria* and *Campylobacter *species [7].

### 2.2. Antimicrobial Activity

The antibacterial activity of enterocin S37 was assessed against the Gram-negative and Gram-positive bacteria listed in Table 1. The cell-free supernatant (CFS) and partially purified enterocin S37, by ammonium sulfate precipitation and two successive reversed phase-high performance liquid chromatography (RP-HPLC) steps, were used in order to assess the antagonism toward the target strains listed in Table 1. *E. faecalis* S37,* E. faecalis* JH2-2, *Lactobacillus brevis *F1.114, *Lb. bulgaricus* 340,* Lb. brevis *F145,* Pseudomonas sp*. 131,* Escherichia coli* CIP 76.24,* Salmonella montevideo, Sal. enterica *serotype *enteritidis* CIP 81.3,* Staphylococcus epidermidis* CIP 68.21, *Bacillus cereus *CIP 78.3,and* Leuconostoc mesenteroides *subsp*. mesenteroides *DSM 20240 were grown on Brain Heart Infusion broth (BHI) medium (AES, Bruz France), whilst* L. monocytogenes *EGDe and* L. innocua *F were grown on Elliker medium (AES). *Campylobacter jejuni* NCTC 11168 was grown in Brucella medium (AES).

The antibacterial activity was measured by the well known agar diffusion test [12]. For this purpose, 100 *μ*L from overnight culture of targeted strains was added to 20 ml of Elliker or BHI soft agar medium already containing 0.8% (w/v) of agar (Biokar diagnostics, Beauvais, France). Wells were performed in solid agar and 50 *μ*l of each sample tested was poured into the wells. The Petri dishes were left at room temperature, in sterile conditions, for 1 h before incubation for 18 h at adequate temperature according to the target strain to be tested. After this period of incubation, the antibacterial activity was detected by observing the inhibition zones around the well containing the CFS. It should be noted that supernatant was obtained by centrifuging at 15000 g during 30 min at 4°C, an overnight culture of *E. faecalis* grown at 37°C for 18 to 24 h, on de Man Rogosa Sharpe (MRS) medium [17] (AES). The CFS was neutralized by addition of 1 N NaOH and filtered through 0.2 *μ*m membrane (Sartorius, Goettingen, Germany).

Arbitrary Unit (AU) was calculated as described by Batdorj et al. [12]. Serial two fold dilution of samples was performed with phosphate buffer (25 mM K_2_HPO_4_, 25 mM KH_2_PO_4_, pH 7) filtered through 0.2 *μ*m membrane (Sartorius). These samples were tested by agar diffusion test on *L. monocytogenes* EGDe strain, as described previously. One arbitrary unit was defined as the reciprocal of the lowest dilution that did not show growth of the target strain.

### 2.3. Bacteriocin Purification

The CFS neutralized and filtered (400 ml) was precipitated with ammonium sulfate to 80% saturated solution. Three distinct fractions were recovered and were resuspended in 40 ml of phosphate buffer (25 mM K_2_HPO_4_, 25 mM KH_2_PO_4_, pH 7); the active one was applied onto RP-HPLC column (ATOLL 15 MP3*;* 250 mm × 10 mm Interchim, Montluçon, France) previously equilibrated with solvent A (0.05%, v/v, trifluoroacetic acid [TFA]). Elution was performed by using a gradient from 0% solvent B (0.045%, v/v, TFA, 80% acetonitrile) to 100% solvent B in 45 min. The flow rate was adjusted to 3 ml.min^−1^ and the absorbance was recorded at 220 and 280 nm. Fractions of 10 ml were collected and tested for their antibacterial activity. The acetonitrile was removed from the active fractions by Speed-Vac concentrator (SC110A, Savant) and pH was adjusted to 6.8 using phosphate buffer (50 mM; pH 7). The active fractions were pooled and the purification of the bacteriocin was continued by RP-HPLC on a Waters Alliance apparatus with Millennium software (Millford, MA, USA). Concentrated bacteriocin (80 *μ*l) was injected into an analytical C_18_ column (Symmetry 300; 5 *μ*m; Spherical 300 Å; 150 × 4.6 mm, Waters, Guyancourt, France) equilibrated in 0% solvent C (0.045% TFA in H_2_O) and 50% solvent D (80% acetonitrile, 20% H_2_O, 0.05% TFA). The elution was performed with a linear gradient from 30% to 100% solvent D in 30 min. The flow rate was adjusted to 0.8 ml min^−1^. The eluted peaks were detected by spectrophotometry measuring the absorbance between 210 and 300 nm with a photo diode array detector (PDA 996; Waters) and collected manually. The fractions were then concentrated in a Speed-Vac concentrator.

### 2.4. Effect of Enzymes, pH and Heat Treatment on Antibacterial Activity of Bacteriocin

The effect of *α*-chymotrypsin (1 mg · ml^−1^), papain (1 mg · ml^−1^), proteinase K (0.5 mg/ml^−1^), lysozyme (2 mg · ml^−1^), and lipase (1 mg · ml^−1^) (all enzymes were from Sigma-Aldrich, Germany) on the antibacterial activity of enterocin S37 was checked by the agar diffusion assay as described by Batdorj et al. [12]. CFS was treated with the aforementioned enzymes for 1 h at 37°C. The pH stability was studied as described by Line et al. [2]. Thus, samples (CFS) of 10 ml were adjusted to pH values ranging from 3 to 10 with 1 N HCl or 1 N NaOH. The obtained fractions were incubated for 2 h at 37°C. After this period of incubation, the antibacterial activity of each treated sample was determined by the agar well diffusion assay [12]. The stability of enterocin S37 was studied at high temperature by heating 100 *μ*l of supernatant at 80°C for 15 min, 80°C for 1 h and 90°C for 15 min. After cooling in ice, each sample was tested for its antagonism by agar well diffusion assay [12].

### 2.5. Sodium Dodecyl Sulfate (SDS) Polyacrylamide Gel Electrophoresis (PAGE)

In order to determine the apparent molecular mass of enterocin S37, the SDS-PAGE gel was cut into two parts. The part of the gel containing the molecular markers and the samples were stained by silver nitrate coloration method [18], and the remaining part, containing only samples, was extensively washed with regularly replaced sterile MilliQ water. Second part of the gel was used for direct detection of antimicrobial activity by overlaying with soft agar (0.8%) seeded with indicator strain *L. monocytogenes *EGDe and incubated overnight at  37°C.

### 2.6. Development of Bacteriocin-Resistant Variants to Enterocin S37

Resistant variants to enterocin S37 were developed by stepwise culture of strain of *L. monocytogenes* EGDe at 30°C in tryptic soy broth (Biokar diagnostics) supplemented with 0.6% (w/v) yeast extract (Organotechnie SA, La Courneuve, France) and called TSYE broth (TSYEB). Enterocin S37 was added at 2- 4- 6- 8- and 10-fold the minimal inhibitory concentration (MIC). The MIC was determined for *L. monocytogenes* EGDe according to Naghmouchi et al. [19] using microplate reader ELx808 (BioTek Instruments, Bad Friedrichshall, Germany). The stability of the resistant variants was checked over 50 generations. After growth of 50 generations in TSYEB at 30°C, the strain was inoculated in the TSYEB containing enterocin S37 at 10-fold MIC.

### 2.7. Effect of K^+^ Channel Modulators

*L. monocytogenes* EGDe and its bacteriocin-resistant variant were grown at 30°C for 24 h in TSYEB in the presence of enterocin S37 at 5 *μ*g · ml^−1^and K^+^ channel modulators such as pinacidil (Pi) at 10 *μ*g · ml^−1^, cromakalim (Cro) at 10 *μ*g · ml^−1^ and glipizide (Gli) at 10 *μ*g · ml^−1^ Combination of enterocin S37 with each of K^+^ channel modulator was also realized in similar conditions. All these K^+^ channel modulators were obtained from Sigma-Aldrich (Germany). Growth was monitored by measuring the optical density at 595 nm (OD_595_) of 250 *μ*l of freshly inoculated medium placed into microplate wells [19].

The inhibitory activity (IA) of enterocin S37 in presence or absence of each K^+^ channel modulator was calculated as described by Naghmouchi et al. [19]. Clearly, a percentage of IA = 100 – 100 (OD_595_(x)/OD_595_(i)) (during exponential growth). The value (x) was the culture containing inhibitor and (i) was the uninhibited control culture. Data were expressed as the percent change in IA obtained with the enterocin S37/K^+^ channel modulator.

## 3. Results

### 3.1. Spectrum of Activity of Enterocin S37

CFS of *E. faecalis* S37 displayed activities against *E. faecalis* JH2-2, *Lb. brevis *F1.114, *Lb. brevis *F145, *L. monocytogenes *EGDe, and* L. innocua *F. However, none antibacterial activity was detected against the remaining strains listed in Table 1. Surprisingly, the anti-*Campylobacter* activity previously observed [7] was not recovered in this study arguing on instability trait (Table 1). Furthermore, the activity observed against strains of *Enterococcus* genus is interesting in order to generate novel knowledge on immunity proteins and cross-resistance within this genus.

### 3.2. Effects of Enzymatic and Physicochemical Treatments on Enterocin S37 Activity

CFS of *E. faecalis* S37 displayed activity against the target strains *E. faecalis* JH2-2, *Lb. brevis *F1.114, *Lb. brevis *F145,* L. monocytogenes *EGDe and* L. innocua *F even after treatments with lipase (1 mg · ml^−1^) and lysozyme (2 mg · ml^−1^). The antibacterial activity observed upon lipase and lysozyme treatments was similar to that observed with the untreated supernatant used as positive controls. However, treatment with proteolytic enzymes, such as *α*-chymotrypsin, papain, and proteinase K resulted in the loss of the bacteriocin activity (Table 2). The stability of antibacterial activity of enterocin S37 remained intact after heating treatments (80°C for 1 h and 90°C for 15 min) and at pH values ranging from 4 to 9 (Table 2). Attempts to reduce putative disulfide bridge (s) of enterocin S37 were performed with *dithiothreitol* (Sigma, Germany); the experiment was not conclusive because of toxicity of this compound, even at low concentrations, towards the target strains.

### 3.3. Purification of Enterocin S37

**Figure 1:**
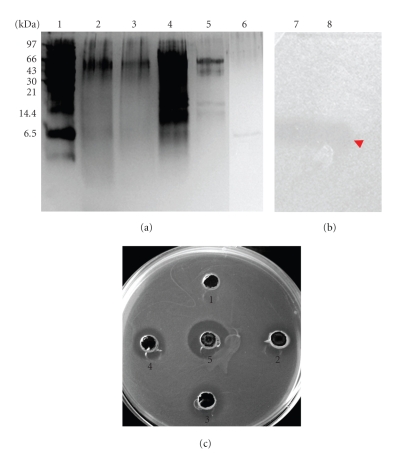
Tricine-sodium dodecyl sulfate-polyacrylamide gel electrophoresis (Tricine-SDS-PAGE). (a) Silver-stained gel. Lane 1: molecular weights markers; lane 2: culture supernatant of *E. faecalis* S37; lane 3: heat and filtered supernatant culture of *E. faecalis* S37; lane 4: active fraction collected after ammonium sulfate precipitation; lane 5: active peak obtained after first RP-HPLC; lane 6: active peak obtained after second RP-HPLC. (b) The gel was overlaid with *L. monocytogenes* EGDe to determine the antimicrobial activity of the purified enterocin S37; lane 7: active peak obtained after first RP-HPLC; lane 8: active peak obtained after second RP-HPLC. (c) Agar diffusion test. Well 1: negative control (phosphate buffer); well 2: heated and filtered supernatant culture of *E. faecalis* S37; well 3: active fraction collected after ammonium sulfate precipitation; well 4: active peak obtained after first RP-HPLC; well 5: active peak obtained after second RP-HPLC.

The bacteriocin was purified by a three-step method including ammonium sulfate precipitation and two RP-HPLC. The first stage consisted in an ammonium sulfate precipitation at 80%, which allowed concentrating the active fraction 10-fold (v/v); subsequently, the active fraction was passed through two successive RP-HPLC columns. Active fractions (those exhibiting antibacterial activity) were eluted at 60% of elution buffer containing 80% acetonitrile, 20% H_2_O, and 0.05% TFA. The purity of each active fraction collected after each step was checked on SDS-PAGE (Table 3, Figure 1).

Bacteriocin appeared to have a molecular weight comprised between 4 and 6 kDa (Figure 1(a)), and fitting thereof with the inhibition zone observed on SDS-PAGE (Figure 1(b)). For this purpose, the electrophoresis gel obtained after migration under nondenaturing conditions was plated directly on Elliker soft agar (0.9%) inoculated with *L. monocytogenes* EGDe with 1% volume transfer (v/v). The other proteins bands revealed by electrophoresis were devoid of antibacterial activity. The antimicrobial activity of each sample, obtained during purification process, was also checked by agar diffusion test on Petri dish (Figure 1(c))

### 3.4. Effect of K^+^ Channel Modulators

**Figure 2:**
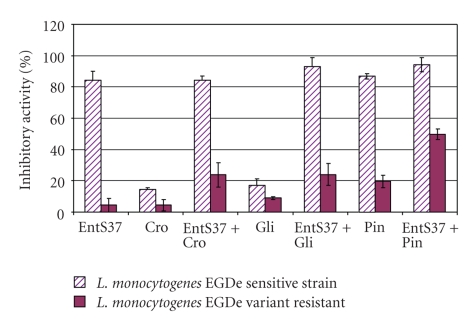
Inhibitory activity of *L. monocytogenes* EGDe sensitive strain and its variant resistant after exposure to enterocin S37 alone (EntS37; 5 mg · ml^−1^) and K^+^ channel modulators alone: pinacidil (Pi; 10*μ*g · ml^−1^), cromakalim (Cro; 10 *μ*g · ml^−1^) and glipizide (Gli; 10 *μ*g · ml^−1^). Values are the mean of three independent measurements.

Both K^+^ channel activators (pinacidil and cromakalim) and inhibitor (glipizide) affected differently the growth of *L. monocytogenes* EGDe and its bacteriocin-resistant variant to enterocin S37 obtained in this research. As shown in Figure 2, the highest effect was attributed to the combination of enterocin S37 (5 *μ*g · ml^−1^) and pinacidil (10 *μ*g · ml^−1^). Effect of the combinations of enterocin S37 with cromakalim (10 *μ*g · ml^−1^) and glipizide (10 *μ*g · ml^−1^) was less important on *L. monocytogenes* EGDe bacteriocin-resistant variant.

## 4. Discussion

Enterococci are known to be widespread in nature. For most enterococcal species, the predominant habitat is the gastrointestinal tract of animals and humans where they can be found in numbers as high as 10^8^ cfu/g of feces [20]. Enterococci were found in foods of animal origin (milk, cheese, and fermented sausages), vegetables and plant materials [21]. Additionally to these natural habitats, they are implicated in nosocomial infections [22].

Many enterococci were reported to produce bacteriocins called enterocins, which are various, having great diversity in their structure, and active against numerous microorganisms, especially foodborne pathogens and against microorganisms of environmental and medical interests [2, 4, 14, 23]. Theppangna et al. [15] have studied a set of 139 strains of *E. faecium* and *E. faecalis* for their capabilities to produce enterocins and they concluded that 51% of isolates were producers of enterocins and 46% of them were able to produce more than one enterocin. The strains producing enterocins were carrying at least one structural gene coding for the most known enterocins (*entA*, *entP*, *entL50AB* and *cylL*) [15]. Other studies pointed out the importance of *E. faecalis* as sources of enterocins [24, 25]. Taken together, these studies indicate the distribution and importance of enterococcal strains. It should be pointed out that most of enterocins so far studied exhibited activity towards Gram-negative and Gram-positive bacteria [8]. We have undertaken isolation and characterization of enterocin named enterocin S37, which is produced by *E. faecalis* recently isolated from chicken feces [7]. Enterocin S37 appeared to be interesting regarding its activities against the food-borne pathogen *L. monocytogenes *EGDe and casually against *Campylobacter jejuni *NCTC 11168 (Table 1). However the anti-*Campylobacter* activity remained unstable and less important than that observed for other bacteriocins recently reported in the literature [2–4]. The data gathered from this study let us think of the possibility of enterocin S37 to be a class IIa bacteriocin, as (i) this peptide was anti-*Listeria*, (ii) has a molecular weight < 10 kDa, (iii) was sensitive to proteases, and (iv) was stable at different pH and high temperatures.

Results of purification indicated that enterocin S37 was highly hydrophobic. The activity increased several folds (from 400 AU · ml^−1^ to 51,200 AU · ml^−1^) during the purification process (Table 3), in parallel with an increase of specific activity, which reaches 1,557.17 AU · *μ*g^−1^. This phenomenon was largely described previously as the result of removing inhibitory compounds [26–28]. The spectrum of partially purified enterocin S37 was the same to that observed with CFS, but with stronger activities (data not shown).

For the first time, the effect of K^+^ channel modulators on enterocin activity was revealed in this research. To this aim, we have utilized two K^+^ channel activators (cromakalim and pinacidil) and one K^+^ channel blocker (glipizide). Cromakalim was shown to act on ATP-sensitive potassium channels and caused membrane hyper polarization, pulls their membrane potential away from the threshold [29]. In the pharmacology sector, the use of cromakalim to treating hypertension was reported [29]. Pinacidil is a cyanoguanidine drug that opens ATP-sensitive potassium channels [30]. Finally, glipizide was described to affect the cell by partially blocking the K^+^ channels, reducing K^+^ conductance and causing depolarization of the membrane, which leads to Ca^++^ ions influx through voltage-sensitive Ca^++^ channels, causing rising of the intracellular concentrations of Ca^++^ ions [31, 32]. Furthermore, the impact of cromakalim and glipizide on growth of sensitive and resistant *L. monocytogenes* strains was studied and resulted to be overall weak. Thus, the inhibition of wildtype (sensitive phenotype) to cromakalim and glipizide was estimated to 14.29% ± 1.38 and 17.14% ± 4.08 while the inhibition of the mutant strain (resistant phenotype) was evaluated to 4.46% ± 3.53 and 8.93% ± 1.06. Pinacidil showed high-inhibitory activity on the wildtype (86.79% ± 1.78) and interestingly not on the mutant strain (19.64% ± 4.02).

Combinations of enterocin S37 and different K^+^ channel modulators suggested an implication of enterocin S37 in a depletion of intracellular K^+^ level, by an action on K_ATP_ channels rather than K_v_ channels. These data are in good agreement with those obtained formerly with nisin A, whose antibacterial activity was connected to K_ATP_ channels with concomitant loss of all intracellular K^+^ and ATP [19]. As for enterocin AS-48 and enterocin P [33, 34], enterocin S37 is certainly responsible of uncontrolled efflux of intracellular K^+^ and affecting sensitive cells growth. The resistant variant was able to grow but was slightly affected by the combination of enterocin S37/K^+^ modulators, except for pinacidil, where the inhibitory effect has reached 49.79% ± 3.36, instead of 23.84% ± 7.73 and 24.11% ± 7.07 when cromakalim and glipizide were added, respectively (Figure 2).

It was also well established that many bacteriocins, including enterocins, interact with membrane lipids leading to the formation of pores and subsequently to the loss of intracellular components, resulting thereof in the cell death [35–39]. The lipid composition of membrane appeared to play an important role in the efficiency of bacteriocin activity [9, 35, 36, 38]. Enterocin S37 alone did not affect the growth of bacteriocin-resistant variant of *L. monocytogenes* EGDe. However, the combination of enterocin S37/K^+^ channel modulators impacted strongly the growth of resistant variant. Otherwise, the K^+^ channel modulators enhanced the inhibitory activity of enterocin S37 on sensitive strain of *L. monocytogenes* EGDe, with values reaching, 92.86% ± 5.90 and 94.29% ± 4.55 when enterocin S37 was combined with glipizide and pinacidil, respectively. Combination of cromakalim and enterocin S37 resulted in 84.29% ±  2.5 inhibition of the growth of sensitive strain; this data is equal to that obtained with enterocin S37 alone 84.21% ± 5.87 (Figure 2). However, in the case of resistant strain, when enterocin S37 was combined to pinacidil, a high-inhibitory activity (49.79% ± 3.36) was observed as compared to combinations of cromakalim + enterocin S37 (23.84% ± 7.73) and enterocin S37 + glipizide (24.11% ± 7.07) (Figure 2).

The mode of action of enterocin S37 could be triggered by potential interaction with cell membrane. The determination of aminoacid sequence of enterocin S37 is under consideration.

## Figures and Tables

**Table 1:** Antimicrobial activities of partially purified enterocin S37.

Strains	Reference	Source and reference	Enterocin S37
*Enterococcus faecalis*	S37	[7]	−
*Enterococcus faecalis*	JH2-2	Laboratory collection	**+**
*Listeria monocytogenes *EGDe	107776	CIP (Paris, France)	**+++**
*Listeria innocua *	F	Laboratory collection	**++**
*Lactobacillus bulgaricus*	340	Laboratory collection	−
*Lb. brevis *	F1.114	Laboratory collection	**++**
*Lb. brevis *	F145	Laboratory collection	**+**
*Pseudomonas sp*.	131	Laboratory collection	−
*Escherichia coli*	76.24	CIP (Paris, France)	−
*Salmonella montevideo*		Laboratory collection	−
*Sal. enterica *serotype *enteritidis *	81.3	CIP (Paris, France)	−
*Staphylococcus epidermidis*	68.21	CIP (Paris, France)	−
*Campylobacter jejuni *	11168	NCTC (London, UK)	−
*Bacillus cereus*	78.3	CIP (Paris, France)	−
*Leuconostoc mesenteroides *subsp*. mesenteroides *	20240	DSM (Braunschweig, Germany)	−
*Streptococcus haemolyticus*		Laboratory collection	−

–: Absence of inhibition,+: Diameter of inhibition zone was less than 3 mm,++: Diameter of inhibition zone was comprised between 3 and 6 mm,+++: Diameter of inhibition zone was higher than 6 mm.

**Table 2:** Effect of enzymes, pH, and heat treatment on antibacterial activity of enterocin S37 against *L. monocytogenes *EGDe and *L. innocua *F.

Treatments	Test strains	
	*L. monocytogenes *EGDe	*L. innocua *F
*Enzymes*		
*α*-chymotrypsin (1 mg · ml^−1^)	−	−
Proteinase K (0.5 mg · ml^−1^)	−	−
Papain (1mg · ml^−1^)	−	−
Lysozyme (2 mg · ml^−1^)	+	+
Lipase (1mg · ml^−1^)	+	+

*Assessment of antibacterial stability of enterocin S37 at different pH values *		
3.0	−	−
4.0	+	+
5.0	+	+
6.0	+	+
7.0	+	+
8.0	+	+
9.0	+	+
10.0	−	−

*Antibacterial activity after heating*		
80°C, 15 min	+	+
80°C, 60 min	+	+
90°C, 15 min	+	+

+: Antibacterial activity detected; *−*: Absence of antibacterial activity.

**Table 3:** Purification of enterocin S37 produced by *Enterococcus faecalis *S37.

Purification step	Volume (ml)	Protein concentration (*μ*g · ml^−1^)	Activity (AU* · ml^−1^)	Specific activity (AU* · *μ*g^−1^)	Purification factor
Filtered culture supernatant	400	10,647.94	400	0.03	1
Ammonium sulfate precipitation	40	5,079.03	12,800	2.52	84
First RP-HPLC	10	56.64	51,200	903.95	30,132
Second RP-HPLC	1	32.88	51,200	1,557.17	51,906

*Arbitrary Unit was calculated as described by Batdorj et al. [12].

## Abbreviations

CIP: Collection of Institut Pasteur (Paris, France)
DSM: DeutschSammlung von Mikroorganismenund Zellkulturen (Braunschweig, Germany)
NCTC: National collection of type strains (London, UK).
